# *Moringa oleifera* Lam. Leaf Peptides: Antioxidant and Antiproliferative Activity in Human Colon Cancer Caco-2 Cell Line

**DOI:** 10.3390/antiox13111367

**Published:** 2024-11-08

**Authors:** Sara Avilés-Gaxiola, Laura Aracely Contreras-Angulo, Israel García-Aguiar, J. Basilio Heredia

**Affiliations:** 1 Health Sciences Department, Universidad Autónoma de Occidente, Blvd. Lola Beltrán and Blvd. Rolando Arjona, Culiacán 80020, Mexico; sara.aviles@uadeo.mx (S.A.-G.); israel.garcia@uadeo.mx (I.G.-A.); 2 Nutraceuticals and Functional Foods, Centro de Investigación en Alimentación y Desarrollo, A.C. Carretera a Eldorado Km 5.5, Col. Campo El Diez, Culiacán 80110, Mexico; lcontreras@ciad.mx

**Keywords:** *Moringa oleifera*, peptides, hydrolysates, cytoprotective effect, antiproliferative activity, cellular antioxidant activity

## Abstract

Reactive oxygen species are produced as part of the cellular metabolism. However, lifestyle can promote an excess in their concentration. Free radicals react with DNA, promoting the appearance of cancer cells. Therefore, natural antioxidants have been suggested as an alternative to prevent this disorder. Peptides are protein fragments that have been produced from various plants. In previous work, *Moringa oleifera* leaf peptides (MOPHs) with antioxidant potential were generated and identified. However, the spectrophotometric methods used to evaluate their antioxidant activity do not fully reflect its potential. In this work, the antioxidant activity of MOPHs was assessed by the ferric reducing antioxidant power assay (FRAP) and cellular antioxidant activity method on the human colon cancer cell line Caco-2. Also, their antiproliferative activity was evaluated. The MOPHs exhibited a FRAP activity of 1435 µmol TE/g, and at 500 µg/mL; the peptides did not show a cytotoxic effect on healthy colon CCD-18Co cells. Moreover, the MOPHs increased Caco-2 antioxidative activity to a greater extent by 73.45% and 83.62% at 250 and 500 µg/mL, respectively. Regarding cellular proliferation, the MOPHs inhibited it by 78.19% and 90.20% at 200 and 500 µg/mL, respectively. These findings highlight the potential of *Moringa oleifera* leaf peptides as functional ingredients with significant health benefits, demonstrating antioxidant and antiproliferative properties.

## 1. Introduction

Reactive oxygen species (ROSs) are produced as a result of cellular metabolism [1]. However, lifestyle can promote their increase, mainly due to exposure to environmental pollution and ultraviolet radiation, tobacco and alcohol consumption, and physical inactivity [2]. When the concentration of free radicals exceeds the capacity of cellular antioxidant systems, homeostasis is affected, and biological molecules are damaged, promoting the appearance of chronic conditions such as inflammatory processes, cardiovascular diseases, neural degeneration, diabetes, and cancer [3]. As for the latter, ROSs react with the genetic material of cells, inducing somatic mutations that, when accumulated, promote the appearance of malignant cells, which have been linked to the onset and development of several types of cancer. In this regard, ROSs also promote the under expression of tumor suppressor genes and the overexpression of oncogenes [4]. Much evidence suggests that the consumption of antioxidants prevents the neoplastic initiation stage, so these molecules have been targeted as an alternative for treating and preventing this disease [5]. Moreover, antioxidant studies have been encouraged since medications traditionally used to treat various types of cancer have been shown to be highly toxic, with documented side effects affecting quality of life [5,6]. Also, some cancer cells have acquired resistance to some of these drugs and existing treatments, not being effective enough to reduce the high mortality rate associated with cancer [6].

Regarding the use of antioxidant molecules, natural ones are preferred over chemically synthesized ones. For example, they are easier to obtain, exert low-toxicity or non-toxic effects, and have built-in chirality, which is difficult to achieve when synthesizing molecules [7]. Also, it has been widely reported that antioxidants of natural origin modulate cell proliferation and death processes [8]. Natural antioxidants are obtained from cereals, legumes, fruits, vegetables, etc., and have been classified as phenols, flavonols, tannins, alkaloids, and peptides, among others [9]. Peptides are protein fragments containing 2 to 20 amino acids that are released from proteins by using digestive enzymes or other proteases.

The specific bioactivity of peptides depends upon their particular amino acid sequence and length [10]. As for their antioxidant activity, they may act as reducing agents, metal chelators, singlet oxygen quenchers, hydrogen donors, etc., and they also inhibit enzymes involved in free radical production [11]. According to what has been reported in various studies, peptides with anticancer potential are mainly those that have antioxidant and antiproliferative activity [12]. The main advantage of these natural antioxidants is that they can directly attack cancer cells without affecting normal ones [13]. Colorectal cancer has been one of the most used models for peptide studies since although enterocytes absorb free amino acids as well as di- and tripeptides through the Ppt1 HfI/peptide cotransporter, these cells can only absorb 0.1% of longer peptides, with the majority going to the large intestine [14].

Many bioactive peptides with antioxidant and anticancer potential have been produced from various plant materials like *Moringa oleifera* (MO). The MO tree is native to the Himalayas and is now widely distributed worldwide, with the African continent taking more advantage of its benefits [15]. MO leaf peptides have shown antidiabetic, antihypertensive, antibacterial, antifungal, and anti-osteoporotic potential [16,17,18,19,20]. The antioxidant effect of MO peptides has also been previously evaluated by DPPH, ABTS, and ORAC [21]. However, more methodologies can be used to assess their antioxidant potential. For example, cellular antioxidant activity is a biologically representative method [22]. This test has been used to predict the antioxidant activity of various molecules of plant origin but less so for hydrolysates. The only existing study of the cellular antioxidant activity of *Moringa leave* peptides was carried out in HepG2 cells [23]. However, nothing is known about the anticancer activity of peptides from MO leaves. The present study is a continuation of previous investigations in which fourteen MO leaf peptides were produced, extracted, and identified (LAYKPPG, YHSEVPV, WPPTFEQPK, LLGFDNR, QVWPTPGLK, FTKDDEWSCFPF, VEQNLVPGLK, TMMLMT, VQLPGWRVFP, SYLPPLSAEVTAK, TMKGPPDTLQ, MPWHEQ, LTAPGQATLPT and LLTPEGPKE). These peptides showed remarkable antioxidant and anti-inflammatory effects [20]. Because it is crucial to learn more about the mechanism of action of antioxidant molecules such as peptides in cancer cells, this work aimed to evaluate more biological properties of peptides obtained from MO leaves. The antioxidant activity of the peptides was assessed by the ferric reducing antioxidant power (FRAP) assay and cellular antioxidant activity method on the human colon cancer cell line Caco-2. Their antiproliferative activity was also evaluated in Caco-2. Previously, the maximum concentration at which the peptides did not exert a toxic effect was determined on healthy colon CCD-18Co cells.

## 2. Materials and Methods

### 2.1. Materials

The chemical reagents used for the protein extraction, hydrolysis, cellular antioxidant assay and the FRAP reagents used were as follows: tris(hydroxymethyl)aminomethane hydrochloride (TRIS-HCl), NaCl, poly(vinylpolypyrrolidone) (PVPP), phenylmethanesulfonyl fluoride (PMSF), ethylenediaminetetraacetic acid (EDTA), ammonium sulfate, pepsin, HCl, NaHCO_3_, NaOH, pancreatin, dichlorodihydrofluorescein diacetate (DCFH-DA), 2,2′-azobis(2-methylpropionamidine)dihydrochloride (APPH), AcONa·3H_2_O, 2,4,6-Tri-(2-pyridyl)-s-triazine (TPTZ), Trolox and FeSO_4_·7H_2_O. In vitro toxicology assay (TOX-7) and 3-(4,5-dimethylthiazolyl-2)-2,5-diphenyltetrazolium bromide (MTT) kits were purchased from Sigma-Aldrich (St. Louis, MO, USA). Cell culture reagents were purchased from GIBCO (Waltham, MA, USA). Human colorectal adenocarcinoma cells (Caco-2) and human colorectal fibroblast cells (CCD-18Co) were purchased from the American Type Culture Collection (ATCC, Manassas, VA, USA).

For the plant material, we used 20 *Moringa oleifera* Lam. plants marked from a larger number of plants cultivated in a family-owned farm of Culiacan, Sinaloa, Mexico (24°51037.0″ N/107°12059.6″ W and 86 m above sea level). The farm has sandy loam soil, which was irrigated at regular intervals as required. The samples from selected three-year-old plants were harvested and taken into the laboratory. They were first soaked in gentle commercial detergent for 15 min. They were immediately washed in running tap water, followed by double-distilled water. Samples were further disinfected with chlorine (50 ppm); subsequently, they were double-washed in double-distilled water. The fresh young leaves were excised from the plants. After cleaning, leaves were dried at 40 °C for 72 h in an Excalibur 3526T food dehydrator (Sacramento, CA, USA). The dried leaves were milled in a KRUPS Gx41011 coffee grinder (CDMX, Mexico). MO leaf flour was stored at 4 °C in sealed containers until further use.

### 2.2. Moringa oleifera Leaf Protein Extraction

MO leaf flour protein was extracted in 0.05 M Tris-HCl buffer pH 8.0 containing 2% (*w*/*v*) PVPP, 0.15 M NaCl, 0.01 M EDTA, and 0.001 M PMSF, 1:5 (*w*/*v*). The solution was agitated at 4 °C for 30 min and filtered through a cheesecloth layer. The solid retained fraction was discarded, and the filtrate was centrifugated at 10,000 rpm and 4 °C for 30 min. The supernatant was precipitated at a 90% saturation of (NH_4_)_2_SO_4_ at 4 °C for 18 h. The precipitate was centrifugated at 10,000 rpm and 4 °C for 30 min. The pellet was recovered, dissolved in double-distilled water, and dialyzed with a membrane with a molecular weight cut-off (MWCO) of 2 kDa against double-distilled water at 4 °C. The dialysate was further lyophilized, and the MO leaf protein (MOP) was stored at −20 °C until further analyses.

### 2.3. Digestion of Moringa oleifera Leaf Protein

MOP was obtained by simulated gastrointestinal digestion [24]. The protein isolate was suspended in pepsin solution (2000 units per mL) in a ratio of 1:20 (*w*/*v*) at 37 °C and pH 3 for 2 h under continuous stirring to simulate passage through the stomach. The pH was adjusted with 1 M HCl every 30 min. Afterward, the pH was adjusted to 7.0 with 1 M NaOH, and subsequently, NaHCO_3_ and pancreatin were added at a concentration of 0.1 M and 100 units per ml, respectively, to simulate intestinal fluid. The solution was stirred for 2 h at 37 °C, and during this time, pH was revised and adjusted with 1 M NaOH every 30 min. Right after, it was placed on ice to stop the reaction, and the enzymes were inactivated by heating the hydrolysate for 20 min at 80 °C. Later, MOP hydrolysate (MOPH) was centrifuged at 4000 rpm and 37 °C for 20 min, and the supernatant was recovered and ultrafiltered with a 5 kDa MWCO to remove enzymes and undigested protein. The permeated product was recovered, lyophilized, and stored at −20 °C until further analyses.

### 2.4. Determination of Antioxidant Activity of Moringa oleifera Leaf Protein and Protein Hydrolysate by Ferric Reducing Antioxidant Power Assay

Antioxidant activity was evaluated using the FRAP method, according to Benzie and Strain, with some modifications [25]. The FRAP solution was made by mixing acetate buffer (400 mM, pH 3.6), TPTZ (20 mM in concentrated HCl), and ferric chloride (60 mM) (10:1:1 *v*/*v*/*v* ratio). For the test, 1 mg of MOP or MOPH was diluted in 1 mL of bidistilled water. An amount of 30 µL of these solutions was placed in each microplate well and mixed with 120 µL of the FRAP solution. The microplate was incubated for 4 min at room temperature in the absence of light, mixed at medium speed for 1 min in the microplate reader, and read at a wavelength of 593 nm. Results are expressed as Trolox equivalent micromoles per gram (µmol TE/g).

### 2.5. Cytotoxic Effect of Moringa oleifera Leaf Protein and Protein Hydrolysates on Human Colon Fibroblasts

For the cell culture, CCD-18Co cells were grown in Eagle’s minimum essential medium (EMEM) supplemented with 10% heat-inactivated fetal bovine serum (FBS) and 1% antibiotic–antimycotic in 75 cm^2^ culture flasks and incubated in a humidified atmosphere (5% CO_2_ at 37 °C).

To measure the cytotoxic effect, the TOX-7 assay kit was used according to the recommendations of Sigma Aldrich (St. Louis, MO, USA), which are based on lactate dehydrogenase activity. For this assay, 1 × 10^4^ CCD-18Co cells were seeded in each well in a sterile 96-well microplate. Once adhered, they were treated with 250, 500, 750, and 1000 µg/mL of MOP or MOPH and incubated for 24 h at 37 °C and 5% CO_2_. Samples were read at an absorbance of 490 nm by a microplate reader. The results are expressed as % mortality.

### 2.6. Determination of In Vitro Antioxidant Activity of Moringa oleifera Leaf Protein and Protein Hydrolysate by Cellular Antioxidant Activity

For the cell culture, Caco-2 cells were grown in Dulbecco’s Modified Eagle’s Medium F12 (DMEM/F12) supplemented with 10% heat-inactivated FBS and 1% antibiotic–antimycotic in 75 cm^2^ culture flasks and incubated in a humidified atmosphere (5% CO_2_ at 37 °C).

For the determination of intracellular ROS production, to evaluate the cellular antioxidant activity of MOP and MOPH, the method reported by López-Barrios was performed [26]. In a clear, flat-bottom and black-walled 96-well microplate, 6 × 10^4^ cells/well were seeded. After 24 h, cells were treated with MOP or MOPH at concentrations of 250, 500, 750, and 1000 µg/mL containing DCFH-DA 20 µM. Afterward, cells were incubated for 20 min at 37 °C and 5% CO_2_. The solution was removed, and cells were washed once with PBS. Finally, 100 µL of APPH 500 µM was added to each well except for the negative control and blank. Fluorescence was emitted at 538 nm upon excitation at 485 nm. It was measured every 2 min for 90 min at 37 °C using a microplate reader. Cellular antioxidant activity (CAA) values were calculated using the following equation:

CAA Unit = 1 − (∫SA/∫CA)

∫SA is the integrated area under the sample fluorescence versus time curve and ∫CA is the integrated area from the control curve.

### 2.7. Antiproliferative Effect of Moringa oleifera Leaf Protein and Protein Hydrolysates on Human Colorectal Adenocarcinoma Cells

For cell culture, Caco-2 cells were grown in Dulbecco’s Modified Eagle’s medium (DMEM)/F12 supplemented with 10% heat-inactivated FBS and 1% antibiotic–antimycotic and incubated in a humidified atmosphere (5% CO_2_ at 37 °C).

Antiproliferative activity was measured using the MTT assay according to the supplier’s recommendation (Sigma-Aldrich, USA). Caco-2 cells were plated on a 96-well sterile microplate at a density of 2 × 10^4^ cells/well. Concentrations of 250, 500, 750, and 1000 µg/mL of MOP or MOPH were tested on Caco-2 for 24 h at 37 °C with 5% CO_2_; likewise, 5-fluorouracil (5-FU) at 250 µM was used as the reference drug to compare the effect of the treated cells. The percentage of cell viability was calculated based on the succinate dehydrogenase activity (SDA) as follows:

% SDA = [(absorbance control − absorbance sample)/absorbance control] × 100

The control was cells grown in supplemented DMEM/F12.

### 2.8. Statistical Analysis

Each experiment was conducted in triplicate, and the standard deviations were calculated. Data were reported as mean ± standard deviations and were subjected to analysis of variance (ANOVA) using the statistical software Minitab version 19 from MiniTab Inc. (State College, PA, USA). Means were compared using Tukey’s multiple comparison test at *p* ≤ 0.05.

## 3. Results

### 3.1. Determination of Antioxidant Activity of Moringa oleifera Leaf Protein and Protein Hydrolysate by Ferric Reducing Antioxidant Power Assay

The values of the antioxidant potential of MOP and MOPH determined by the FRAP assay are presented in Table 1. MOP was taken into account in this experiment, and the following ones, since it was considered a positive control and had the purpose of determining if the hydrolysis process, and therefore the generation of peptides, is responsible for bioactivity.

### 3.2. Cytotoxic Effect of Moringa oleifera Leaf Protein and Protein Hydrolysates on Human Colon Fibroblasts

**Figure 1:**
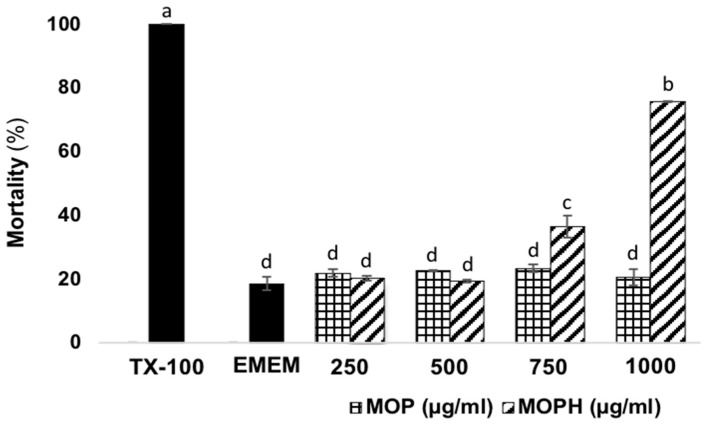
Cytotoxic effect of *Moringa oleifera* leaf protein (MOP) and protein hydrolysate (MOPH) on human colon fibroblasts (CCD18-Co) expressed as % of mortality. Triton X-100 (TX-100) was used as the positive control, considering that it is known to promote cell death. EMEM (cells and medium) contained CCD18-Co cells without treatment to observe normal cell death. Different letters in bars show significant differences (*p* < 0.05).

The cytotoxicity of MOP and MOPH was tested on CCD-18Co to determine a nontoxic concentration in healthy cells (Figure 1). Triton x-100 (TX-100) was used as a positive control, considering that it promotes the maximum release of the enzyme lactate dehydrogenase and therefore the maximum death rate. As a negative control, CCD18-Co cells were grown under the same conditions but without any stimulus. Thus, their death percentage is considered the normal cell death rate. The mortality values of MOPH tested doses 250, 500, 750, and 1000 µg/mL in CCD18-Co were 20.32 ± 0.69, 19.35 ± 0.46, 36.44 ± 3.32, and 75.63 ± 0.11%, respectively. The effect of the first two doses was not statistically different from that of the negative control. Based on this, it is established that these concentrations have no adverse impact on healthy cells’ viability. Furthermore, all MOP tested doses had no adverse effect on CCD18-Co cells’ viability.

### 3.3. Determination of In Vitro Antioxidant Activity of Moringa oleifera Leaf Protein and Protein Hydrolysate by Cellular Antioxidant Activity

**Figure 2:**
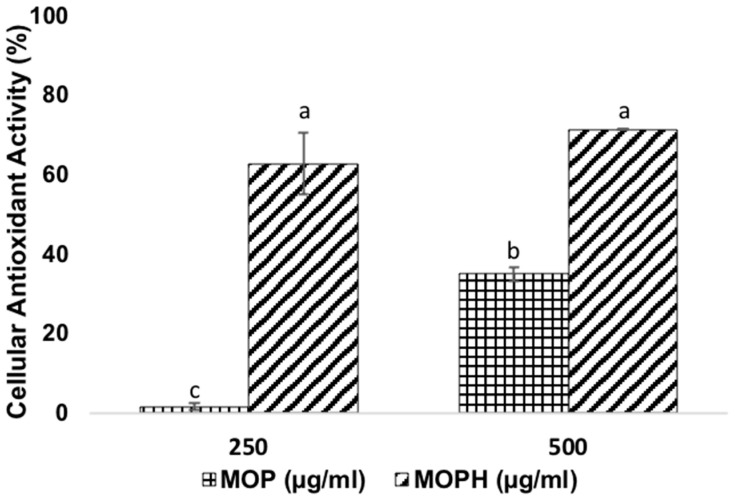
Cellular antioxidant activity as affected by *Moringa oleifera* leaf protein (MOP) and protein hydrolysate (MOPH) in human colorectal adenocarcinoma cells (Caco-2) treated with APPH. Different letters in bars show significant differences (*p* < 0.05).

The antioxidant activity of MOP and MOPH, as assessed by the CAA assay, was determined by measuring the prevention of the oxidation of 2′,7′-dichlorofluorescin diacetate to 2′,7′-dichlorofluorescin through the quenching of the peroxyl radical (RO_2_**·**). The above is reflected in a reduction in cellular fluorescence. The cellular antioxidant activity of MOP and MOPH is presented in Figure 2. When using MOP, the cellular antioxidant activity was increased concentration-dependently. The 250 µg/mL concentration did not produce antioxidant activity on Caco-2 cells; nevertheless, 500 µg/mL increased it significantly by 35.10 ± 1.77%. On the other hand, MOPH increased Caco-2 antioxidative activity to a greater extent by 73.45 ± 8.54% and 83.62 ± 2.92% at the doses 250 and 500 µg/mL, respectively. Although the change between the doses was not statistically significant, a tendency was observed that as the dose increases, the oxidative activity decreases.

### 3.4. Antiproliferative Effect of Moringa oleifera Leaf Protein and Protein Hydrolysates on Human Colorectal Adenocarcinoma Cells

**Figure 3:**
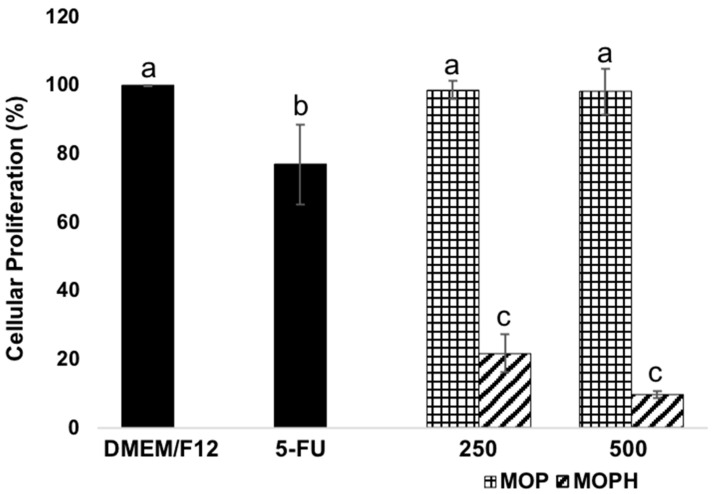
Determination of antiproliferative effect of *Moringa oleifera* leaf protein (MOP) and protein hydrolysate (MOPH) in human colorectal adenocarcinoma cells (Caco-2). Cells grown in Dulbecco’s Modified Eagle’s Medium (DMEM/F12) were used as the negative control, and 5-fluorouracil (5-FU) was used as the reference drug to compare the effects of the treated cells. Different letters in bars show significant differences (*p* < 0.05).

The antiproliferative activity of MOP and MOPH was screened by the MTT assay; cellular proliferation against tested concentrations is shown in Figure 3. Cells were treated with 5-FU since it is the most widely used drug for the treatment of colon cancer, and it is known to be an apoptosis inducer. At the tested concentrations, MOP did not affect Caco-2 proliferation. Briefly, 5-FU inhibited Caco-2 cellular proliferation by 23.10 ± 11.61%. MOPH inhibited Caco-2 cellular proliferation to a greater extent, reducing it by 78.19 ± 5.43% and 90.20 ± 1.09% at the doses 200 and 500 µg/mL, respectively. Although the change between the doses was not statistically significant, a tendency was observed that as the dose increases, the antiproliferative effect increases.

## 4. Discussion

According to what is reported in Table 1, there was a significant difference between the antioxidant activity of MOP and MOPH, which increased by 57% after the hydrolyzing process. The reason may be that when peptide chains are released from complex proteins, amino acid residues are exposed and can interact with free radicals [27]. In the particular case of the FRAP assay, it measures the ability of molecules to serve as reducing agents by donating electrons to the Fe^3+^-2,4,6-tripyridyl-S-triazine complex, converting it to a more stable ion, Fe^2+^ [28]. It was established by the FRAP assay that the amino acids that promote high antioxidant capacity are sulfur-containing amino acids, such as cysteine and methionine. On the other hand, the presence of hydrophobic amino acids in peptide structures, such as isoleucine, proline, glycine, and methionine, contribute to their high electronic density [29].

There is little available information in the literature on determining the antioxidant activity of vegetable hydrolysates using the FRAP assay. Among what has been published that takes Trolox per gram of protein as a reference, hydrolysates from soybean, rice, chickpea, and cassava leaf have antioxidant activities of 15.32, 18.78, 27.31 and 1046.00 TE µmol/g, respectively [30,31,32,33]. Compared to the above reported values, the antioxidant activity of MOPHs is higher than that of legume hydrolysates but very similar to that of cassava leaf. This may be due to the leaves’ particular amino acid profile. RuBisCo is the main leaf protein, and glycine is its most abundant amino acid found in 8 of the 14 peptides that make up MOPHs [34]. As previously discussed, glycine is linked to high FRAP activity [28].

Based on the above, this methodology confirms the antioxidant activity of the peptides obtained from *Moringa oleifera* leaves and shows another mechanism by which they act.

As for peptide safety, it is essential that new molecules studied for their antioxidant activity and ability to prevent and treat cancer cause the least possible damage to the healthy tissues of the human body [35]. In this sense, one of the advantages of peptides is their low toxicity and ability to be used in a large amount. The main reason for this is that the product of their degradation is amino acids that cells can use as nutrients [14]. Also, these peptides are small molecules and have no immunogenicity [36]. The effect of plant-derived proteins and peptides has not been widely evaluated in the CCD-18co. Regarding peptides, various concentrations have been reported not to be toxic for this cell line. These concentrations range between 10 and 800 µg/mL for hydrolysates from plants such as *Phaseolus vulgaris* L. and soybean [37,38]. As for MOPH concentrations, they fall within this range (Figure 1). It has been determined that the toxicity of peptides in healthy mammalian cells depends on the amount that manages to enter them. When peptides are found in high concentrations, they promote the loss of mitochondrial membrane potential, resulting in fragmentation. This phenomenon can also occurs in the cell membrane [39]. In this case, the number of peptides that enter a cell depends on their composition. The main characteristic is the proportion of hydrophobic amino acids in the peptides since when this is >30%, their entry into the cells is facilitated [40]. Regarding the peptides identified and reported in our previous research [20], 11 of the 14 sequences show this characteristic. Regarding proteins, it is established that they are usually not toxic at high concentrations because their entry into cells is limited by their size, which slows down passive diffusion across the plasma membrane, which may be the reason why MOP did not cause CCD-18co toxicity at any of the concentrations tested [41].

Chemical assays are often used to determine the antioxidant activity of various molecules. However, these sometimes do not consider highly important aspects such as cellular absorption. In this regard, cell assays are of great significance [42]. As mentioned, 500 µg/mL of MOP showed an antioxidative activity of 35.10 ± 1.77% in Caco-2. At the same amount, MOPHs increased it by 83.62 ± 2.92%, demonstrating that the hydrolysis process is responsible for this bioactivity. Similar values regarding the reduction in oxidation in Caco-2 have been reported. As for amaranth hydrolysate, 1160 µg/mL led to a significant inhibition of ROS, keeping cells in the basal state [43]. On the other hand, 2.5 mg/mL of canary seed hydrolysate had a cellular antioxidant activity of 80% in Caco-2 [44].

Other investigations have yielded significant values: 1000 µg/mL of soybean hydrolysate decreased the ROS level in H_2_O_2_-stimulated Caco-2 by 22.74%, and 2500 µg/mL of *Phaseolus vulgaris* L. hydrolysates had a cellular antioxidant activity of 73% in Caco-2 treated with the free radical generator ABAP [45,46].

The antioxidant activity of peptides depends on their sequence. Among the amino acids that are most closely related to the antioxidant activity of a peptide are glutamic acid, glycine, alanine, leucine, and phenylalanine. Glutamic acid is a negative amino acid due to an excess of electrons and has quenching activity on free radicals. Due to this property, it also inhibits metal-mediated oxidation [47,48]. Hydrophobic amino acids such as leucine, alanine, and glycine, as mentioned above, promote the passage of peptides into cells. On the other hand, they contain imidazole rings, which are proton donors [49]. In particular, leucine reduces Fe^3+^, and has a long aliphatic side chain that interacts with the acyl chains of fatty acids [50]. As for phenylalanine, its aromatic ring functions as a proton donor. The tyrosine phenolic hydroxyl group also acts as a hydrogen donor, being able to quench radicals. This amino acid also scavenges the peroxyl radicals generated during the AAPH assay. It has been associated with high CAA values mainly because it has a higher ability to remove peroxyl radicals than other amino acids [51,52]. It has been reported that peptides containing tyrosine have twice the antioxidant activity of those without it in their structure [53]. Three of the fourteen peptides previously identified have this amino acid within their structure [20].

Due to their high antioxidant activity, plant-derived peptides are among the most recently studied molecules as an alternative for the treatment or prevention of cancer. These molecules offer various advantages, such as a large number of sources, few or no side effects, and high specificity and efficiency [35,54]. As for MOPH, this is the first research that reports its effect on cancer cells.

Briefly, 5-FU at 250 µM reduced Caco-2 cellular proliferation by 23.10 ± 11.61%. This value is low compared to what has already been reported, mainly because the action of 5-FU is time-dependent and begins to affect cell proliferation more strongly until after 48 h [55], unlike MOPHs, which reduced Caco-2 cellular proliferation to a greater extent, reducing it by 78.19 ± 5.43% and 90.20 ± 1.09% at 200 and 500 µg/mL doses, respectively, in 24 h. Chickpea hydrolysate at these concentrations (500 µg/mL) reduced Caco-2 cell proliferation by 45% [56]. At 300 µg/mL, a soybean peptide reduced Caco-2 cell proliferation by around 70% [57]. Higher amounts (650 µg/mL) were required for walnut hydrolysates to exert a similar antiproliferative effect on Caco-2 [40]. Quinoa protein hydrolysate reduced Caco-2 proliferation by 51.45% at a concentration of 8 mg/mL [58].

Regarding the antiproliferative activity of MOPH, it has been established that this may be due to the size of the peptides, which allows them to penetrate cell membranes and interact with oncogenic proteins inside cells and with surface receptors, which can promote arrest of the cellular cycle [59]. A type of amino acid that influences the antiproliferative capacity of peptides in cancer cells is one that is positively charged, like lysine and arginine. Peptides with a net positive charge bind to the membranes of cancer cells (which have a net negative charge), generating pores that affect cell integrity [60]. Three of the fourteen identified peptides have a net positive charge [20]. Other amino acids important in peptides with anticancer activity are proline, histidine, and tryptophan. As for proline, it increases peptide flexibility [61]. Histidine gives anticancer peptides the ability to induce cancer cytotoxicity by membrane permeability. Tryptophan has been found to enter cancer cells by the endocytic pathway and binds to the major groove of nuclear DNA [62]. Eleven of the fourteen identified peptides have at least one of these amino acids [20].

MOP did not affect Caco-2 proliferation, which might be because protein structures find it hard to pass through cell membranes due to their size and polarity. Due to the above, a small amount or none reaches the cell’s interior [63].

## 5. Conclusions

Our findings show that peptides obtained from the protein extracted from *Moringa oleifera* leaves have antioxidant potential that is not only limited to chemical assays but also those involving human cells, specifically cancer cells. In addition, their antiproliferative potential in cancer cells is becoming evident for the first time, thus opening up a space for investigating these molecules as preventive or treatment agents in a disease that significantly impacts the global mortality rate. This is of great importance since it is essential to consider that one of the greatest challenges for medicine today is finding an effective cancer treatment, and these types of scientific studies reinforce the idea that there are molecules of natural origin that can help manage this disease. As for peptides, the diverse structures that have been identified unfold a range of possibilities for their study and application, having the potential to be used as pharmaceutical products. However, more studies are needed to verify their beneficial effects.

The results presented in this research paper are promising and provide helpful information. These molecules could be studied together or separately in different cancer models. On the other hand, they could be included as part of the formulation of different foods to improve consumers’ quality of life. More assays, especially in vivo evaluation, are essential to confirm their safety and efficacy.

## Figures and Tables

**Table 1:** Antioxidant capacity assessed by the ferric reducing antioxidant power assay.

Sample	FRAP (µmol TE/g)
MOP	912.5 ± 0.032 ^b^
MOPH	1435 ± 0.035 ^a^

Different letters show significant differences (*p* < 0.05).

## Data Availability

The data presented in this study are available upon reasonable request from the author (S.A.-G.).
